# A Computerized Bioinspired Methodology for Lightweight and Reliable Neural Telemetry

**DOI:** 10.3390/s20226461

**Published:** 2020-11-12

**Authors:** Olufemi Adeluyi, Miguel A. Risco-Castillo, María Liz Crespo, Andres Cicuttin, Jeong-A Lee

**Affiliations:** 1 Ministry of Communications and Digital Economy, Federal Secretariat, Abuja 900001, Nigeria; olufemi.adeluyi@commtech.gov.ng; 2 Engineering Physics, Department of Science, National University of Engineering, Av. Tupac Amaru 210, Cercado de Lima 15333, Peru; mrisco@accesus.com; 3 Multidisciplinary Lab, International Centre for Theoretical Physics, Via Beirut 31, 34100 Trieste, Italy; mcrespo@ictp.it (M.L.C.); cicuttin@ictp.it (A.C.); 4 Department of Computer Engineering, Chosun University, 309 Pilmun-Daero, Dong-Gu, Gwangju 61452, Korea

**Keywords:** telemetry, compressed sensing (CS), electroencephalogram (EEG), personalized health monitoring, virtual instrumentation

## Abstract

Personalized health monitoring of neural signals usually results in a very large dataset, the processing and transmission of which require considerable energy, storage, and processing time. We present bioinspired electroceptive compressive sensing (BeCoS) as an approach for minimizing these penalties. It is a lightweight and reliable approach for the compression and transmission of neural signals inspired by active electroceptive sensing used by weakly electric fish. It uses a signature signal and a sensed pseudo-sparse differential signal to transmit and reconstruct the signals remotely. We have used EEG datasets to compare BeCoS with the block sparse Bayesian learning-bound optimization (BSBL-BO) technique—A popular compressive sensing technique used for low-energy wireless telemonitoring of EEG signals. We achieved average coherence, latency, compression ratio, and estimated per-epoch power values that were 35.38%, 62.85%, 53.26%, and 13 mW better than BSBL-BO, respectively, while structural similarity was only 6.295% worse. However, the original and reconstructed signals remain visually similar. BeCoS senses the signals as a derivative of a predefined signature signal resulting in a pseudo-sparse signal that significantly improves the efficiency of the monitoring process. The results show that BeCoS is a promising approach for the health monitoring of neural signals.

## 1. Introduction

There is a steady rise in the number of patients with neurological disorders, and it has become the leading cause of deaths and disability-adjusted life-years [1]. This, along with a decline in global healthcare budgets, [2] has led to a rapid increase in research activities that aim to use personalized health monitoring systems to continuously monitor neural and other physiological signals within the comfort of the patient’s home [3]. Electroencephalograms (EEG) and electrocorticograms (ECoG) are the main neural signals that are monitored in these efforts.

Medical instruments have a strict requirement for dependability because inaccurate measurements can have fatal effects. In addition to the need for dependability, personalized health monitoring (PHM) systems also need to be portable and need to utilize algorithms that are resource-light, in terms of complexity, latency, and power. Medical virtual instruments (MVIs) are a ready fit for such systems because they utilize a hardware–software codesign approach that enables a large portion of the medical instrument to be implemented in software [4].

EEG signals are usually monitored with multichannel systems that may have up to 256 channels. This can result in a very large dataset that takes up significant storage space and consumes considerable power for the sensing, processing, and transmission phases. The power consumed can easily drain the battery of a PHM system.

Compressive sensing (CS) is an emerging paradigm for energy-efficient sensing, data compression, and transmission of biosignals [5]. The CS approach involves the transformation of the sensed data into a domain where the data is sparse in order to reduce the signal processing requirements. Unfortunately, neural signals are nonsparse in the time and transformed domains. As such, it is difficult to directly apply the CS algorithms to them. Block sparse Bayesian learning bound optimization (BSBL-BO) was proposed and has been widely accepted as an efficient CS approach for sensing neural signals [6]. Science and engineering often draw inspiration from nature, [7,8] and for this paper, we sought to draw a similar inspiration for the sensing and telemetry of neural signals.

In this work, our approach is modelled as a biosystem with two MVIs that imitate a weakly electric fish both as the signal source and the sensing MVI. Our approach, known as bioinspired electroceptive compressive sensing (BeCoS), is based on the active electroception sensing approach used by the fish. A signature signal is transmitted and a remote object is sensed by detecting a pseudo-sparse signal that is a differential with respect to the signature signal. Our technique, CS, and BSBL-BO are described in further detail in Section 2.

The BSBL-BO and BeCoS techniques are compared with respect to coherence, latency, compression ratio, power, and structural similarity. The coherence compared the frequency content of the original and transmitted signals and BeCoS showed an improvement of 35.38% when compared to BSBL-BO. The latency examined the processing time of both methods and BeCoS was 62.85% faster. The compression ratio showed the capacity of both techniques to sparsify the EEG signal, while the power metric analyzed the energy consumption. BeCoS showed an improvement of 53.26% for the compression ratio, and it utilized 13 mW less in processing each epoch of EEG data. The structural similarity accessed the capacity of each technique to transmit signals that are visually similar to the original signals. The result from BeCoS was 6.295% worse than BSBL-BO but the signals were still strikingly similar to the original values. Further details are given in Section 4.

## 2. Materials and Methods

### 2.1. Compressive Sensing

Traditional sensing techniques are based on the Nyquist theorem, [9] which requires signals to be sampled at a frequency equal to at least double their bandwidth in order to perfectly capture the signal. Compressive sensing (CS) techniques approach the challenge of sensing in a much different manner.

In CS, the goal is to find a base, **γ**, to transform high-dimensional analogue data, ***x***, of dimension N, into a k-sparse analogue dataset x* using

(1) $$x=\gamma x^{*}.$$

The next step in CS involves the use of a user-defined sensing matrix **Φ** to generate ***y***, a compressively sensed representation of x using Equations (1) and (2).

***y*** = **Φ*x***. (2)

The original signal ***x*** is then reconstructed at the remote receiver using ***y*** and **Φ**. ***y*** is a compressively sensed representation of **x** when **γ** and **Φ** are incoherent. This condition is met when **Φ** is chosen as a random matrix. The CS approach is effective when there is a sparse representation of the original signal. In this approach, the greater the level of sparsity of the sensing matrix **Φ**, the greater is the potential compression ratio and the lower are the energy requirements.

CS provides a good option for the lightweight sensing, processing, transmission, and reconstruction of signals. However, CS systems need to use signals that are sparse in the time or transformed domains in order to reconstruct the signals with a high level of fidelity. EEG signals are not sparse in either the time or transform domains, which makes the traditional CS-based EEG telemonitoring approach inadequate for clinical applications.

For nonsparse signals, it is necessary to use a sparsifying dictionary matrix ***D*** to obtain a sparsified form of x ($x^{d}$) as shown in Equation (3). Equation (2) can thus be replaced by Equation (4).

(3) $$x\;=\;Dx^{d}$$

(4) $$y=\Phi Dx^{d}.$$

Again, the success of the approach depends on the system’s ability to find a sparse representation of ***x***. This underscores the importance of finding an appropriate user-defined dictionary matrix ***D***. This process can be challenging for arbitrary signals such as EEG [10] because general dictionary matrices are unable to sparsify them sufficiently.

The CS approach is increasingly being used as a sensing approach in different domains [10,11,12,13]. For example, the Random Triggering-Based Modulated Wideband Compressive Sensing (RT-MWCS) is based on random modulation at randomly selected points and used for sparse multiband signals [10]. A number of methods are also used to sparsify signals, including the use of Discrete Cosine Transform (DCT) and Discrete Wavelet Transform (DWT) sparsifying dictionaries.

Spatiotemporal Sparse Bayesian Learning (STSBL-EM) sparsifies signals by learning the joint correlation structure in the multichannel signals, exploiting the linear and nonlinear dependency between blocks [12]. Ref. [11] also used block sparse techniques to compress accelerometer readings of stroke victims. CS is useful where sensors are limited, measurements are expensive, sensing is time consuming, and power is constrained.

### 2.2. Block Sparse Bayesian Learning Bound Optimization

The block sparse Bayesian learning (BSBL) framework has been proposed as a lightweight approach for processing biosignals in a manner that avoids the requirement of an optimal dictionary matrix. It is based on the premise that most natural signals have a rich structure and can be represented as a concatenation of blocks [14] as shown in Equation (5). It uses user-defined block partitions and assumes that most of these blocks are sparse.

(5) $$s=\;{\left[\underset{s_{1}^{T}}{\underbrace{s_{1},\ldots ,s_{l_{1}}}},\ldots ,\;\underset{s_{g}^{T}}{\underbrace{s_{l_{g-1}+1},\ldots ,s_{l_{g}}}},\;\right]}^{T}$$

where s_i_ describes the block interval i of length l.

BSBL-BO is a fast-learning BSBL based on the bound-optimization method. It has been effectively applied as a lightweight solution for the reconstruction of temporally correlated EEG signals [6]. The authors’ empirical results showed that nonsparse EEG signals can be reconstructed with a high level of fidelity using BSBL-BO with standard dictionary matrices.

In Ref. [6], the solution was based on the use of a sparse binary matrix as the sensing matrix **Φ**, and an inverse discrete cosine transform (DCT) as the dictionary matrix ***D***. The use of this type of dictionary matrix created a system that was 2.58 times faster than a system that did not use the dictionary matrix [6]. BSBL-BO has been used as the reference technique for comparing BeCoS.

### 2.3. The Basics of Electroception

Electroception, also known as electroreception, refers to the ability to perceive natural electrical stimuli. Lampreys, bees, sharks, cockroaches, and rays are examples of species that use electroception either for communication or for identifying the location of objects [15]. Some of these species are highly sensitive to changes in the electric field around them. For example, the shark can detect changes as low as 1 × 10^−10^ V/m [16].

Electroception can either be active or passive. In passive electroception, the sensing species detects a field originating from another species, whereas in active electroception, the species senses its environment by generating an electric field and detecting the distortions made to it by the field of another animal or object. The biological sensors used for the detection are known as the ampullae of Lorenzini. The generated electric field, also known as its signature signal, may be modulated to make it unique to the sending species. It can give information of the fish’s individual identity, sex, and social and nonsocial behavior [17].

**Figure 1:**
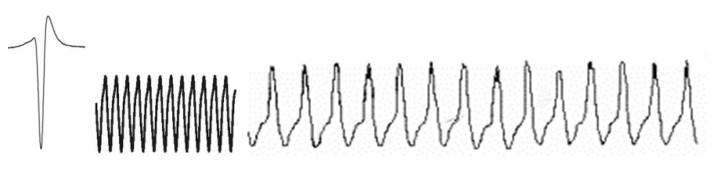
Examples of signature signals used for electroception.

The weakly electric fish is a good example of an animal that uses active electroception [18,19]. This approach can either be based on small pulses (pulse-type) or a quasi-sinusoidal discharge (wave-type), and it allows the species to identify the properties of the sensed object. For example, a weakly electric fish can detect and discriminate objects based on their ohmic and capacitive properties [20,21]. The signature signal of the fish has *very large intraspecific waveform variability and usually has a triangular or sawtooth pattern* [22]. Figure 1 shows some examples of signature signals.

Similar electrolocation techniques are used by bats and in radar technology. In those approaches, there is a Doppler shift owing to the relative motion between the source and the target. However, in electroception, there is *no frequency shift* due to the movement of electric signals [23].

The fish makes the process of sensing more efficient by *matching the impedance* between itself and its sensing environment. In this manner, it can use a minimal amount of energy to sense its environment. The impedance Z is a combination of the resistance R and the capacitive reactance X_C_, as shown in Equation (6). All living organisms have considerable capacitive properties that filter the signature signal in a manner that maintains the phase [24].

Z = R + X_C_. (6)

The resistance is independent of frequency, whereas the capacitive reactance is inversely proportional to the product of the frequency and capacitance. The fish detects the distortions to the electric field that reflect the conductivity of the sensed object. The change is measured as a *change in the transepidermal voltage gradient* in the area next to the skin of the prey. It was also noted that good conductors increase the transepidermal voltage, while insulators have a decreasing effect. Further, *the detection of object waveforms relies on a purely temporal (and not spectral frequency) analysis* [25].

A description of the key features of electroception used in BeCoS is given in the section on “Porting Key Electroceptive Features into BeCoS”.

### 2.4. Bioinspired Electroceptive Compressive System (BeCoS) Methodology

**Figure 2:**
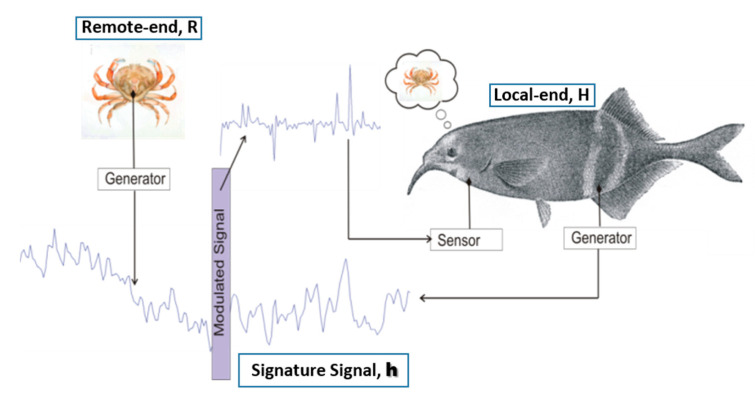
Bioinspired electroceptive compressive sensing (BeCoS) sensing approach.

The BeCoS approach utilizes the electroreceptive approach for the sensing, compression, and transmission of physiological signals, as shown in Figure 2. The approach is based on the wave-type active electroception option. The modulated vector derived from this approach contains a high proportion of elements whose amplitude is close to zero. Section 4 provides further details.

BeCoS assumes that the signature signal vector **h** (of length E) originates from H, analogous to the local end of the weakly electric fish. The **h** vector is transmitted in the direction of R, analogous to the position of the object to be sensed, which generates an **r** vector (of length Z). Z >> E and Z = wE (where w is a positive integer).

A modulated vector **p** is generated when the **h** and **r** vector waves interact. With j as the current epoch being processed and *w* as the total number of epochs, the vector **p** is given by

(7) $$p_{j}=\left[p_{1},\;p_{2},\;p_{3},\ldots ,\;p_{w}\right]={\left[\frac{dr_{1,\;}dr_{2,\;}\ldots ,dr_{w,}}{dh}\right]|}_{j=1,2,3,\ldots ,\;w},$$

where each vector **p*_w_*** has a length E, which is the size of each epoch of the neural signals.

In this approach, dβ/dα is a numerical differentiation given as the gradient between two adjacent points and defined as

(8) $$\frac{\mathrm{df}\left(\alpha \right)}{d\alpha }\;=\;\frac{f\left(\alpha +\Delta \alpha \right)-\;f\left(\alpha \right)}{\left(\alpha +\Delta \alpha \right)-\;\left(\alpha \right)}.$$

The neural signals of interest are usually measured and stored as time series data. As such, there is a need to compute the modulated signal **p** = d**r**/d**h** using differentiation-by-parts.

(9) p=drdh=[drdt]/[dhdt+δ]=r˙(h˙+δ)δ={1 × 10−6, h˙=00, h˙≠0

where **δ** is a small positive stabilizer that prevents numerical instability when $\dot{h}=0$.

Similarly, **r** is reconstructed by integration as shown in Equation (10). The cumulative trapezoidal numerical integration method was used.

(10) $$r_{recon}=\;\int_{0}^{w}p\dot{h}\mathrm{dt}=\int_{0}^{w}pdh\approx \;\frac{w}{2}\left(p_{0}+2p_{1}+2p_{2}+2p_{3}+\ldots +2p_{w-1}+p_{w}\right).$$

BeCoS is based on the analogy of electroception in weakly electric fish, where the fish sends out stimuli (known as a signature signal) to its prey and senses the properties of this prey by modulating the prey’s electric field with respect to this signature signal. In a similar vein, BeCoS senses and processes the EEG signal of interest using a signature signal and modulating the sensed EEG signal with respect to the signature signal. The properties, design, and choice of the signature signal are discussed in Section 3.3 and Section 3.4.

## 3. System Model and Performance Metrics

**Figure 3:**
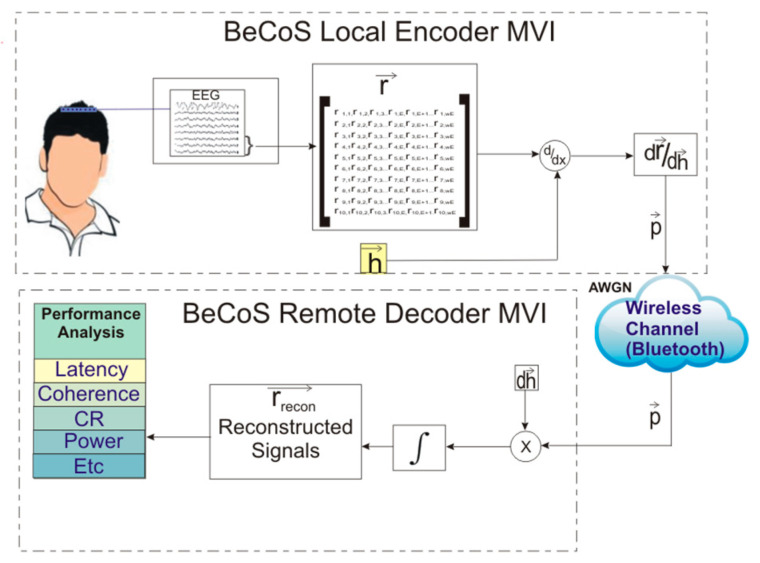
Conceptual diagram of the BeCoS system model.

The BeCoS model is shown in Figure 3. The multichannel physiological signals are captured in real time using appropriate sensors at the patient’s end—the local end of the MVI. A sparse differential signal is then generated in quasi-real time. For each channel, a vector (**h**) of length E is selected as the signature signal and all subsequent signals are differentiated with respect to the appropriate signature signal.

This differential signal (**p**) is wirelessly transferred to the physician’s end—the remote MVI. Here, the differentiated signal is integrated with respect to d**h** in order to reconstruct the original signals. An additive white Gaussian noise (AWGN) channel with an SNR of 0 dB is assumed. The reconstructed signals (**r_recon_**) are then compared to the original signal using a set of performance metrics.

The **r** vector is segmented into w, equal nonoverlapping segments, each representing an epoch block, with a length equal to the length of the signature signal vector **h**:

(11) $$r=\left[\underset{1\mathrm{st}\;\mathrm{seg}}{\underbrace{u_{1},\ldots ,u_{E}}},\underset{2\mathrm{nd}\;\mathrm{seg}}{\underbrace{u_{E+1},\ldots ,u_{2E}}},\ldots ,\underset{\left(w-1\right)\mathrm{th}\;\mathrm{seg}}{\underbrace{u_{E\left(w-2\right)+1},\ldots ,u_{\left(w-1\right)E}}},\underset{\mathrm{wth}\;\mathrm{seg}}{\underbrace{u_{E\left(w-1\right)+1},\ldots ,u_{\mathrm{wE}}}}\right],$$

where u_i_ is the ith element of **r**.

### 3.1. Dataset of EEG Signals Used

Standard EEG databases have been used for this study. The EEG dataset signals were taken from a 64-channel EEG motor movement/imagery dataset. It was obtained from an arrangement of sensors based on the international 10–10 system and provided on the PhysioNet website [26]. It contains over 1500 one and two-min EEG recordings. The signals were sampled at 160 samples/s, and the following eight channels were used for the analysis—Fc5, Fc3, Fc1, Fcz, Fc2, Fc4, Fc6, and C5, as channels 1–8 respectively. Each channel had a sequence length of 9760 data points. An epoch length E of 244 was used, resulting in 40 epochs (w).

**Figure 4:**
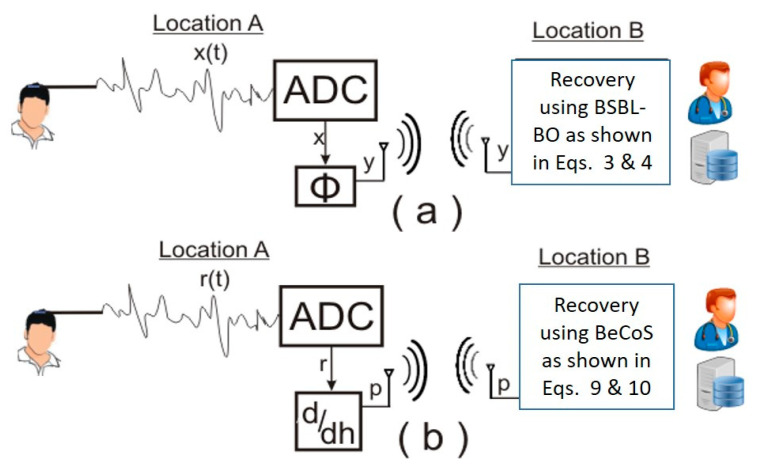
System diagram for testing models: (**a**) block sparse Bayesian learning-bound optimization (BSBL-BO) and (**b**) BeCoS.

**Figure 5:**
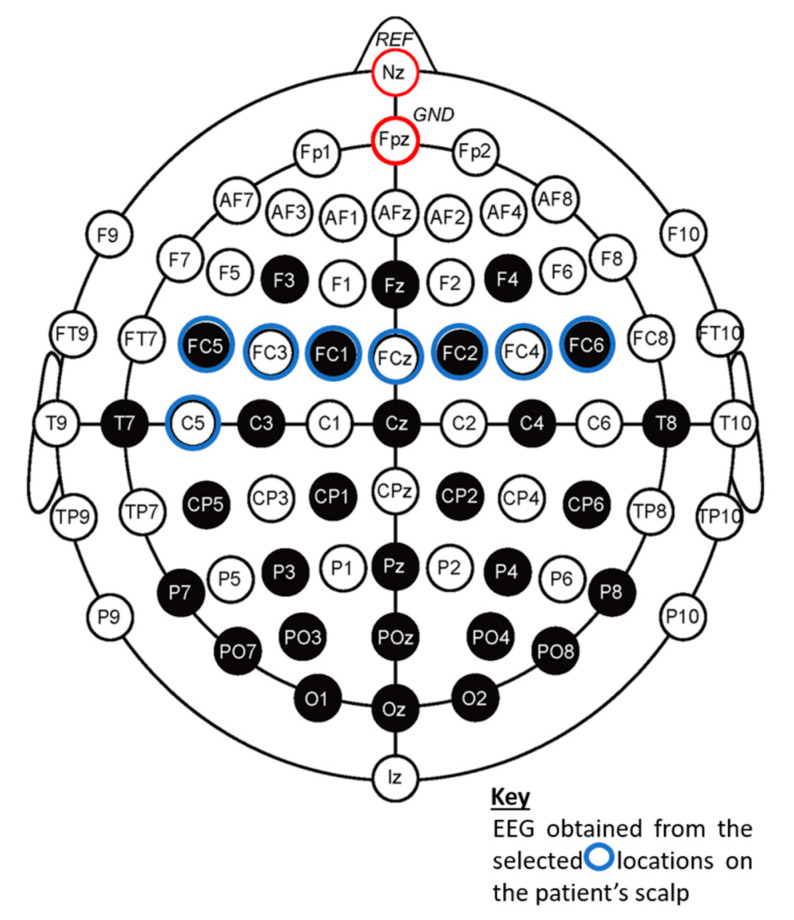
Arrangement of electrodes used to obtain the electroencephalogram (EEG) signals used in the analysis.

The signals were used to simulate a typical signal compression, transmission, and reconstruction scenario for EEG signals as shown in Figure 4. EEG signals, generated from the electrodes attached to a patient’s scalp, are fed into an analog to digital converter (ADC) to be processed by a computer and this process emulates the signal acquisition phase. Location A refers to the patient-end of the MVI, where resource-constrained portable and mobile instruments are used. The instruments are usually powered by batteries. By contrast, location B refers to the physician-end of the MVI, where the signals are reconstructed on desktop computers or servers and powered by AC power. Figure 5 shows the arrangement of electrodes that was used to obtain the EEG signals used in the analysis.

### 3.2. Performance Metrics

Five performance metrics have been chosen to evaluate the BeCoS approach. They are coherence, latency, compression ratio (CR), power, and structural similarity, as described in this section. We ran the experiments on MATLAB (version R2012a) installed on a desktop computer PC with a 3-GHz CPU and 2 GB RAM. The computer had a 32-bit Windows XP Professional operating system. The power consumption was calculated using Xilinx Power Analyzer after running both techniques on a Xilinx Virtex 5 Field Programmable Gate Array (FPGA)-(XUPV5-LX110T).

#### 3.2.1. Coherence

EEG signal processing techniques rely on the frequency content of the signal [27,28,29]. As such, it is important to take the frequency content into consideration when comparing the signals at the local and remote ends. Spectral coherence provides this type of comparison. It is a measurement of the linear correlation between two neural electrodes [30] as a function of frequency; this is useful for comparing EEG signals [31]. It computes the amount of synchrony between two stationary signals at a specific frequency. The coherence metric compares the signals sensed at location A of Figure 4 with the signals reconstructed at location B of the same figure. The magnitude squared coherence, C_XY_, of two signals X and Y is given by

(12) $$C_{\mathrm{XY}}\left(f\right)=\frac{{\left|P_{\mathrm{XY}}\left(f\right)\right|}^{2}}{P_{\mathrm{XX}}\left(f\right)P_{\mathrm{YY}}\left(f\right)}.$$

P_XX_ and P_YY_ are the power spectral densities of X and Y, respectively. P_XY_ is the cross spectrum of X and Y. It is calculated based on the standard Welch’s averaged periodogram method [32]. A Hamming window size of 125 was used for the computation.

#### 3.2.2. Latency

The latency is calculated as the time between the acquisition of the sensed signal at location A and its reconstruction at location B of Figure 4. A zero delay over the noiseless wireless channel has been assumed. The learning phase of the BSBL-BO algorithm takes place at location B, prior to reconstruction. Many PHM systems require a near-real time monitoring in order to enable the physicians to continually monitor the biosignals of patients remotely. It is important to ensure minimum latency for the processing and transmission of these signals to the physician. [33].

#### 3.2.3. Compression Ratio

The compression ratio (CR) refers to the ratio of the dimensionality of the original signal to that of the sparsified sensed signals. The dimensionalities (nonsparseness) of the sensed and original signals are given as m and n, respectively. CR is given by Equation (13). The compression takes place at location A, as shown in Figure 4.

(13) $$\mathrm{CR}=1-\frac{m}{n}.$$

#### 3.2.4. Power Consumption

Power consumption is a major consideration for health monitoring systems because they are likely to depend on batteries for their energy requirements [6,34,35]. According to Ref. [13], low-energy health monitoring systems are lighter and more comfortable for patients because they use smaller, longer lasting batteries. Power is important at the patient end (location A) because of the use of batteries; however, it is not an important consideration at location B. The patient-side core-engines of BeCoS and BSBL-BO were implemented in a Xilinx Virtex 5 FPGA and used to calculate the power consumption.

#### 3.2.5. Structural Similarity

The frequency content of EEG signals has already been identified as an important feature for neural signal processing. The second important feature is the structural similarity of the reconstructed waveform with respect to the original signal [36]. A structural similarity (SSIM) index [6] is the standard metric for quantifying this feature. However, it has been shown in Ref. [36] that even a slight translation, scaling, or rotation of the reconstructed signals can greatly affect the SSIM value. The authors of Ref. [36] went further to propose a complex-wavelet SSIM (CW-SSIM) as a more robust metric for comparison. The CW-SSIM index has been chosen as the metric because the BeCoS approach scales and translates the signals. CW-SSIM is calculated as

(14) $$\tilde{A}\left(s_{x},s_{y}\right)=\frac{2\left|\sum_{i=1}^{N}s_{x,i}s_{y,i}^{*}\right|+j}{2\sum_{i=1}^{N}\left|s_{x,i}+{\;s}_{y,i}^{*}\right|+\;j},$$

where s_x_ and s_y_ are the coefficients extracted from the same spatial locations of the same wavelength sub-bands of the two images being compared; s* is the complex conjugate of s. j is a small positive constant (stabilizer).

### 3.3. Porting Key Electroceptive Features into BeCoS

This section shows how BeCoS integrates the most relevant features of the electroception discussed in Section 2.3. Some of these features were italicized for emphasis in that section. The features are identified and discussed below.

(i) Same type: The signature signal should be of the same type as the monitored signal. This requires the following:
  - The signal should be of the same length as the signal being monitored. In the case of long-term monitored signals, BeCoS requires the biosignal to be monitored in segments equal to the length of the signature signal
  - The signal should be a similar biosignal to the biosignal of interest. As such, an EEG signal will be used for monitoring EEG signals
  - More importantly, there should be a good level of impedance matching between the signature signal and the segments of the biosignal of interest. This feature is further described below in feature (iv).
(ii) Signal sparsification: The signature signal of the electric fish significantly sparsifies the monitored signals. BeCoS also uses a similar signature signal to achieve sparsification without the need for the base or dictionary matrices shown in Equations (1)–(4). The level of sparsity can be further increased through the design of a signature signal with very large gradients at specific points as determined by the designer.
  As shown in Equation (9), the modulated sparse signal **p** is computed by dividing the intersignal gradient of the sensed signal by that of the signature signal. As such, the designer can generate a very sparse signal, by choosing a signature signal $\dot{h}$ with many large swing gradients (those that are very large compared to the corresponding gradient of the sensed signal $\dot{r}$).
(iii) Support for sample rate conversion: The design of a signature signal described in (ii) above can also be used for the downsampling of the sensing process and for sampling rate conversion without the need for the measurement matrix used in standard CS techniques. By noting the points in the signature signal where there are very large gradients, the sensing protocol can avoid sampling those points because **p** will always be close to zero at those points.
  For example, the sampling rate can be halved by ensuring that the signature signal has very large swings at every other point or one-third of the sampling rate can be used if two out of every three points have large swings.
(iv) Impedance matching: As mentioned previously, the weakly electric fish uses an impedance matching between the local and remote ends in order to ensure an efficient sensing process. As shown in Equation (15), this impedance has both ohmic (R) and reactive (X_C_) components. The “Basics of Electroception” section highlights the fact that electroreception is not affected by shifts in frequency, and it maintains the phase of the signature frequency.

Z = R + X_C_. (15)

This suggests that the impedance matching in electroception aims to maintain the ohmic value, while keeping the frequency-dependent reactance at a minimum. The transfer function of a system is a mathematical function relating the output or response of a system to the input or stimulus [37]. For systems that have the same impedance, the transfer function is equal to 1. This appears similar to a scenario where input and output EEG signals are matched. In such a scenario, the coherence is also equal to 1. As such, for BeCoS, we will use a coherence term to represent the ohmic resistance.

The capacitive reactance X_C_ for capacitance C at frequency f is computed [38] as

(16) $$X_{C}=\frac{1}{2\pi \mathrm{fC}}.$$

The capacitance C can be calculated as

(17) C=IdVdt

(18) $$C=\varepsilon_{o}\varepsilon_{r}\frac{A}{d},$$

where I is the current in A, dV/dt is the voltage swing rate in V/s, εr is the dielectric constant, and ε_o_ is the permittivity of free space (≈ 8.854 × 10^−12^ F/m). A is the overlap area between the two objects and d is the distance (in m) between the two objects.

The brain can be modeled as a constant current source [39] and in electroception, the overlap area between the fish and the prey is relatively constant. The dielectric constant and the permittivity of free space do not change. As such, only d and dV/dt may vary; these are the parameters that can be adjusted to alter the capacitance. Both terms need to be low in order to maintain a high C. This is logical because the fish would prefer sensing prey located at a close distance to it. The EEG signals do not provide any d parameter, but the voltage swing rate can be calculated as

(19) $$Swing=\frac{1}{t}\sum_{0}^{t}\left|x_{t}-x_{t-1}\right|,$$

where x_t_ is the EEG signal at time t.

The swing ratio (SR) is the ratio of the voltage swing of the system response (EEG signal being sensed) to the voltage swing of the stimulus (signature signal). The impedance equation shown in Equation (15) can be rewritten to indicate the parts for coherence and SR as shown in Equation (20). BeCoS does not work with impedance per se; therefore, another term is used to describe this similarity: zygosity (Z_BeCoS_). The name was inspired by a similar term used to describe the degree of identity in the genomes of twins [40].

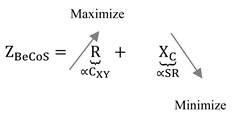
(20)

Z_BeCoS_ depends on the coherence and SR:

(21) $$Z_{\mathrm{BeCoS}}\propto \left(C_{\mathrm{XY}},\;\mathrm{SR}\right).$$

From Equations (20) and (21), an ideal optimized signature signal for BeCoS should have the following properties:

(i) A high level of coherence to the sensed signal
(ii) A low SR (i.e., the voltage swing of the signature signal should be high when compared to the voltage swing of the original EEG signal)

The second property can be attained using a signature signal that follows a pattern similar to the pattern of the natural signature signal used by weakly electric fish, i.e., the sawtooth waveform pattern. However, this signal still needs to be coherent with the sensed signal in order to meet the first requirement.

According to Ref. [41], it is possible to maintain a high level of coherence between two signals with different amplitudes and/or phases as long as the phase difference tends to remain constant. In calculating coherence, it is necessary to know how stable the phase between two waves is and how quickly it changes with time. In Ref. [42], the coherence of a high-order harmonic spectrum was controlled by adjusting the sawtooth shape parameter.

EEG signals reflect the personalized information about the unique anatomical and functional attributes of a patient’s brain, including personality traits and genetic information [43,44,45]. The signal coherence of EEG signals was used as a biometric in a recent study [30]. As such, the signature signal should have a high coherence when compared to a signal segment taken from the measured EEG signal.

On the basis of the foregoing discussions, a procedure for designing an ideal optimized signature signal is presented in the next section.

### 3.4. Designing an Optimized Signature Signal

**Figure 6:**
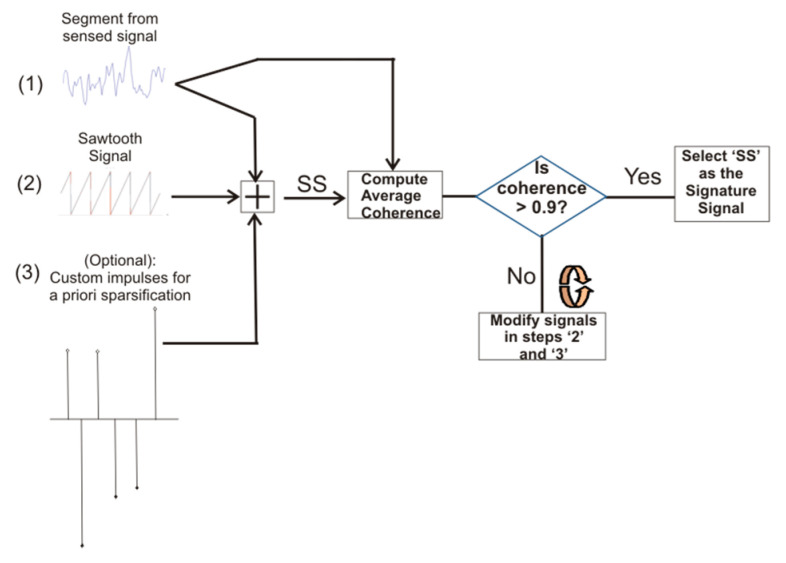
Steps for generating an optimized signature signal.

This section describes the process for designing an ideal optimized signature signal that satisfies the primary requirement of high coherence and the secondary requirement of a large swing ratio as described in the previous section. The process is shown in Figure 6.

The following are the steps for generating an ideal optimized signature signal for BeCoS:

(i) The first option for obtaining a nonoptimized signature signal involves the selection of a signal that is highly coherent with the signal to be sensed. A segment of the sensed signal can be used as the seed signal. In this study, the first epoch is used as the seed signal.
(ii) The second option for a more optimized signature signal requires the addition of a sawtooth signal with a known voltage swing to the coherent signature signal used in the first option. This composite signal should still be coherent to the measured signal.
(iii) A third option for an ideal optimized signal requires the addition of high-value input pulses to the summation of the seed signature signal and sawtooth signal. The output of step (iii), called as SS, is then compared with the seed signal to determine the coherence. If the average coherence is high enough (≥0.9), then SS is chosen as the signature signal. If the coherence is less than 0.9, steps (ii) and (iii) are iteratively modified until an SS with the required coherence is attained. This gives an ideal optimized signature signal but the process has extra computational requirements.

## 4. Results

The EEG datasets were used to compare BSBL-BO and BeCoS on the basis of the five metrics described in the previous section. The tests were conducted on a Xilinx Virtex 5 FPGA and an Intel Pentium 4 system, with a 3.0-GHz CPU and 2 GB RAM. For the BSBL-BO experiments, a 122 × 384 sparse binary sensing matrix **Φ** was used, with a 244 × 244 inverse DCT matrix as the dictionary matrix ***D***.

### 4.1. Coherence

**Figure 7:**
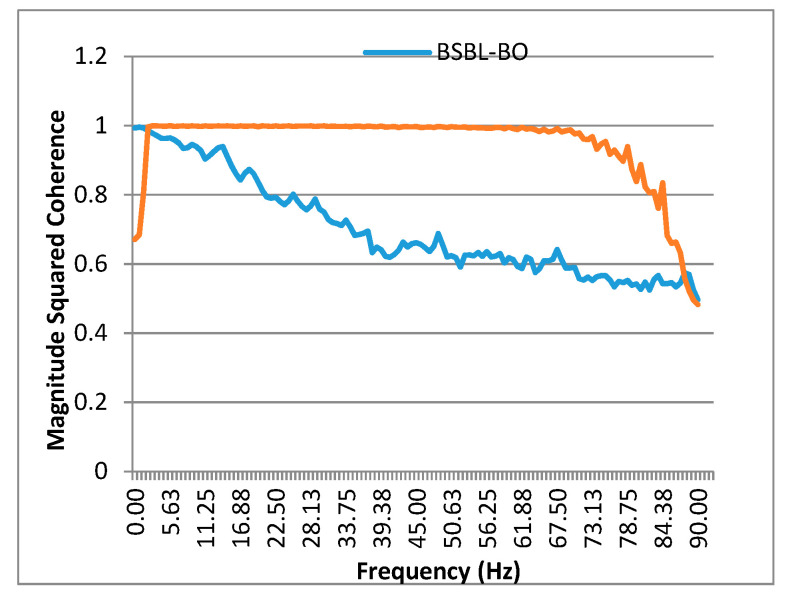
Coherence across the frequency spectrum.

The average coherence value for BeCoS and BSBL-BO were compared (Table 1). The average coherence was also compared across a frequency range of 0–90 Hz (Figure 7). BeCoS had an average coherence of 0.949 across the eight channels, whereas BSBL-BO had an average coherence of 0.701. In addition, BeCoS maintained near-optimum coherence between 2.1 and 84.38 Hz. On the other hand, BSBL-BO fluctuated across the spectrum with a peak of 0.996 at 0.7 Hz and a trough of 0.497 at 90 Hz.

Overall, BeCoS had 35.38% better average values. BSBL-BO is an adaptive learning approach that exploits the intrablock correlation of the signals. This is less linear than the BeCoS approach and increases the power of the noise in the reconstructed signal. BeCoS generates a reconstructed signal that is linearly dependent on the original signal because the differential and integral operators used have a linear time invariant property in the Fourier domain.

**Figure 8:**
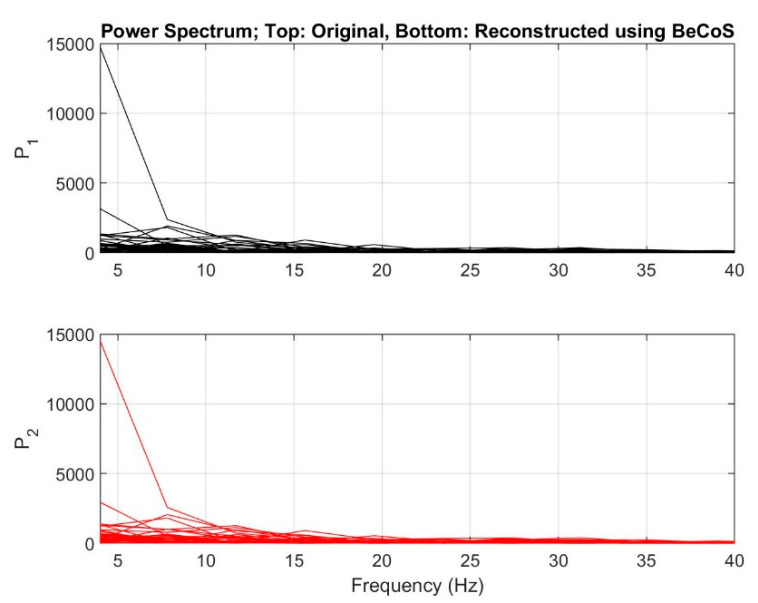
Power spectral density for original and reconstructed signals.

We also obtained the power spectrum for the original signals and those reconstructed with BeCoS. The results showed that signal power across the frequencies for the original signal was consistent with the signal power level for the reconstructed signal across the given frequencies. Some diagrams are shown in Figure 8.

### 4.2. Latency

Latency refers to the time taken for sensing, processing, transmitting, and reconstructing the neural signals. In BSBL-BO, it involves these stages as well as the learning stage, as discussed earlier. For BeCoS, it involves the steps shown in Equations (9) and (10). The sensing, processing, and reconstruction phases of BeCoS essentially involve a few additions and multiplications. For BSBL-BO, it involves additions on the accumulators that result from the sparse binary sensing matrix, the multiplications with the inverse DCT dictionary matrix, and the learning process.

With BeCoS, we had an average of 0.117 ms for processing each epoch, compared to 5.869 ms for BSBL-BO. As such, BeCoS used only 1.99% of the processing time used by BSBL-BO. The processing time we obtained was similar to the time reported in the reference BSBL-BO paper [6], where it took 105 ms to process an epoch. The epoch length used in that paper was 384 data points for 32 channels. This results in 0.273 ms per data point or 2.085 ms per epoch. The paper also used a 192 × 384 sparse binary matrix to sparsify the signal, whereas this study used a 122 × 244 sparse binary matrix. By factoring in this ratio of 2.477, one can estimate that the process described in the reference paper would have taken approximately 5.165 ms/epoch for the experimental setup used in this study. In addition, the PC used in the reference paper had a 2.8-GHz CPU and 6 GB RAM, whereas this study used a PC with a 3-GHz CPU and 2 GB RAM. This may also explain why the result in the reference paper was slightly faster than the BSBL-BO processing results obtained in this research.

### 4.3. Compression Ratio

By representing the sensed signal with respect to a prespecified signature signal, the BeCoS approach reduces a large portion of the original values to values that are close to zero. Values of the sensed signal x, where |x| < 0.99 mV, were taken to be sparse. This pseudo-sparsity reduces the dimensionality of the transmitted signal and increases the effective CR (Equation (13)). The comparison was based on the same optimal configuration for BSBL-BO used to attain a low-cost EEG system in Ref. [6] (CR of 50%). As seen in Table 2, BeCoS achieved an average CR of 76.73% or 53.26% better than BSBL-BO, even though the nonoptimized signature signal was used for the analysis.

### 4.4. Power and Hardware Resource Consumption

As stated earlier, previous research already shows that weakly electric fish use the electroceptive approach to maximize the energy efficiency of their sensing process, and we have also used it for the efficient sensing, processing, and transmission of the neural signals. We implemented the core compression engines of BeCoS and BSBL-BO in hardware in order to obtain a realistic estimation of the power consumption at the patient end of the telemonitoring system.

The EEG data was stored in a ROM with a resolution of 16 bits. To mimic the original sampling process, at each rising edge of the clock, one EEG signal was transferred to the internal buffers. The BSBL-BO-based compression was expressed as shown in Equation (2) (***y*** = **Φ*x***), with each column containing only two nonzero entries to allow the use of accumulator registers, rather than multipliers. The system has buffers to receive all the EEG signals for each epoch because it requires them to determine the final content of the result buffers. The buffers were implemented using configurable logic blocks (CLBs). The resulting output signals were then generated on the basis of the sensing matrix.

For both designs, we used our simple bus architecture (SBA) [46]. The SBA is lightweight computer architecture that implements a minimum essential subset of the open-source Wishbone signals interface [47]. It consists of software tools and intellectual property (IP) cores that are interconnected by buses, enabling the implementation of a system on chip (SoC). The availability of the essential blocks enables the system designer to concentrate on the core design, helping to speed up the design process and making it portable across different FPGA platforms. FPGA-based systems support design flexibility and adaptability [48].

The BeCoS compression engine also receives the EEG signal from the ROM. For BeCoS, a numerical differentiation is computed and only two successive values are required to compute this. As such, the BeCoS engine has three internal buffers. The first two store the successive EEG signals, whereas the third computes the numerical difference and transfers to the results buffer after dividing by the appropriate signature signal value for that location.

The systems were implemented on a Xilinx Virtex 5 FPGA (XUPV5-LX110T). The resource utilization for both systems is shown in Table 3 for the flip flops (FF), look-up tables (LUTs), input-output blocks (IOBs), and block RAMs (BRAMs). The value change dump (VCD) was used with the Xilinx Power Analyzer to estimate the power consumption of both systems as shown in Table 3.

BeCoS used 13 mW less power than BSBL-BO to process one epoch of EEG signals at the patient’s end. To put this figure in better perspective, let us assume that the system at the patient’s end is running on a Samsung Galaxy 4 phone with a Li-ion battery rated at 2100 mAh and 3.8 V. This battery would serve the BSBL-BO system for 8837 h and the BeCoS system for 8933 h—a difference of 96 h or 4 d.

### 4.5. Structural Similarity

The average CW-SSIM values for both BSBL-BO and BeCoS are shown in Table 4.

Table 4 shows that BeCoS has an average CW-SSIM decrease of 6.295%. As noted in the metrics section of structural similarity, the SSIM is highly altered by the scaling of the signal. Similarly, its enhancement (CW-SSIM) is still affected by scaling and rotation, even though it is more robust than SSIM. There is a degree of scaling in the BeCoS approach as a result of the numerical integration during the reconstruction phase, and this invariably lowers the CW-SSIM.

**Figure 9:**
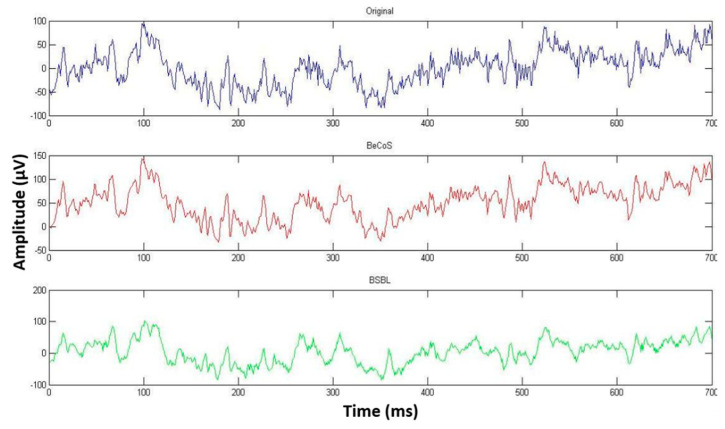
Complex-wavelet structural similarity (CW-SSIM) for Channel 7 [1:700].

**Figure 10:**
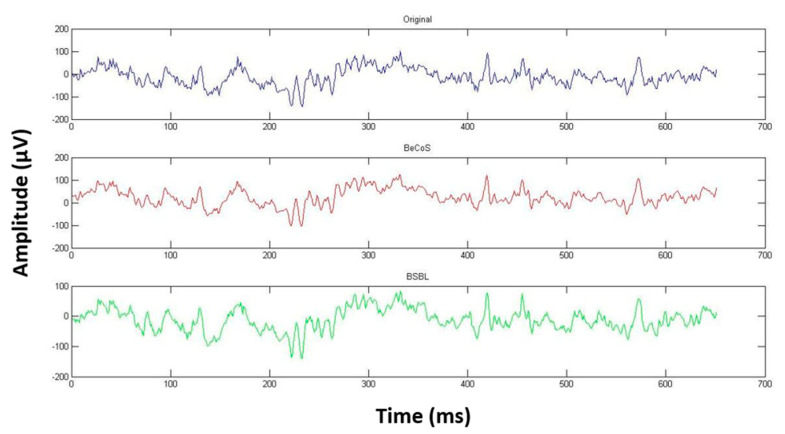
CW-SSIM for Channel 1 [3000:3650].

**Figure 11:**
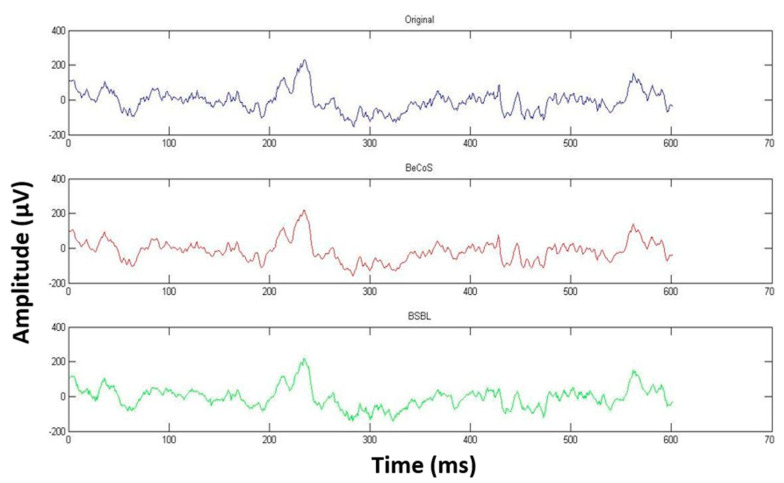
CW-SSIM for Channel 4 [6300:6900].

However, as seen in Figure 9, Figure 10 and Figure 11, the original, BeCoS, and BSBL-BO figures show that regardless of the scaling and translation, the structure of the reconstructed signal using the BeCoS approach is still strikingly similar to the original EEG signal.

#### Effects of Variations on SSIM and CW-SSIM

To further explain the lower CW-SSIM value for BeCoS, we consider a random sample shown in Table 5 with columns for the original sample, its numerical differentiation, numerical integration, numerical integration plus the offset value, and the variation. Numerical integration, as opposed to analytical integration, uses approximations and introduces a nonuniform variation when compared to the original signals. In order to use analytical approaches, the exact equation of the signal must be known in advance, which is not possible for real-time physiological signals. As such, a numerical integration method is the most feasible integration approach that can be used for such signals.

**Figure 12:**
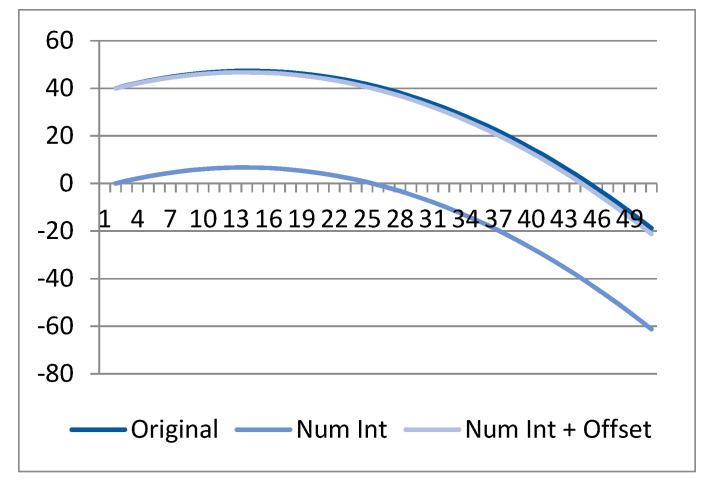
Effect of numerical methods on signal reconstruction.

Figure 12 shows the original sample and sample reconstructed by numerical integration as well as this reconstructed sample value plus an addition of the initial offset.

**Figure 13:**
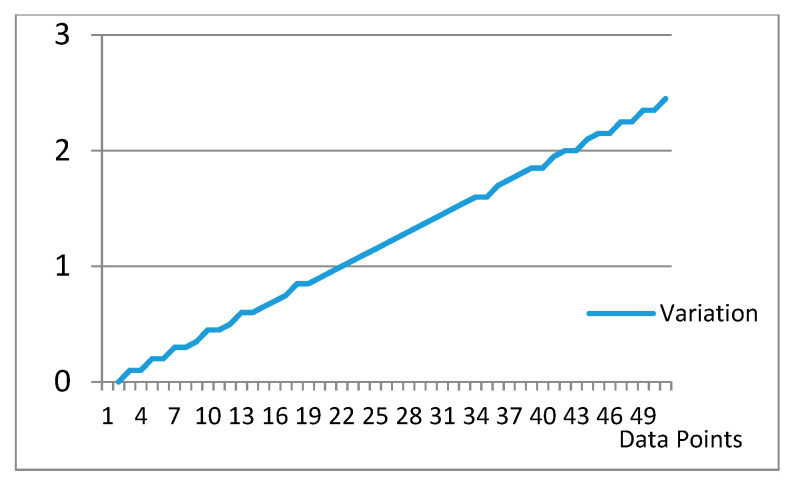
Model of the variation generated by numerical methods.

The figure clearly shows that all three signals have a similar structure. The variation is shown in Figure 13. A regression analysis was used to generate a linear regression variation (LRV) model in order to estimate the equation of this variation. The following equation was obtained:

*y* = 0.049011*x* + (−0.02478) (22)

We used the data in channel 1 of the EEG signals used in this study to visualize the effect of variations on the SSIM and CW-SSIM values. The SSIM and CW-SSIM were calculated by comparing the data in channel 1 with the following samples:

(i) Original sample (comparing it with itself, Or)
(ii) Original sample minus 1 mV (Or − 1)
(iii) Original sample plus 1 mV (Or + 1)
(iv) Original sample minus 5 mV (Or − 5)
(v) Original sample plus 5 mV (Or + 5)
(vi) Original sample plus 10 mV (Or + 10)
(vii) Original sample plus 20 mV (Or + 20)
(viii) Original sample plus 50 mV (Or + 50)
(ix) Original sample plus linear regression variation model (Or + LRV)
(x) Original sample plus linear regression variation model, treating it one epoch at a time (Or + LRV, 1 epoch)

**Figure 14:**
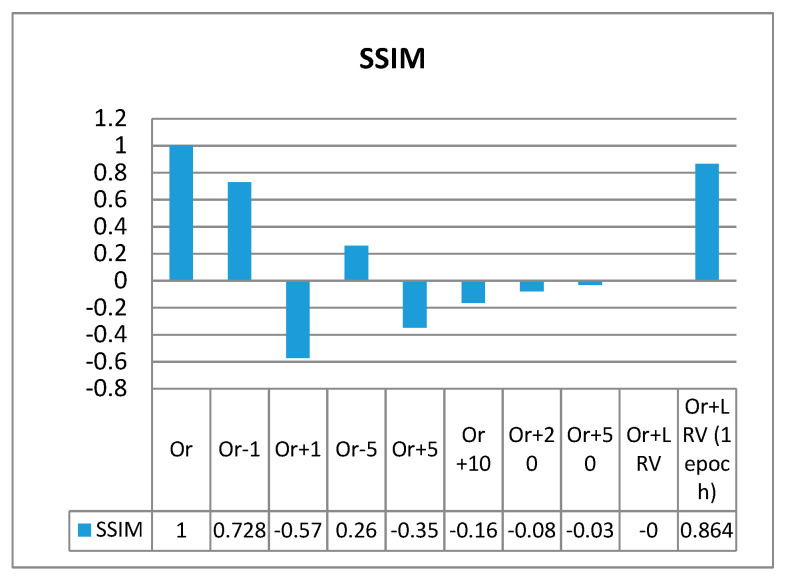
The effect of variation on SSIM.

As seen from Figure 14, any variation beyond 1 mV significantly diminishes the SSIM values. The SSIM value for Or + LRV is almost zero, but it gives a better result when it is treated epoch by epoch. 

**Figure 15:**
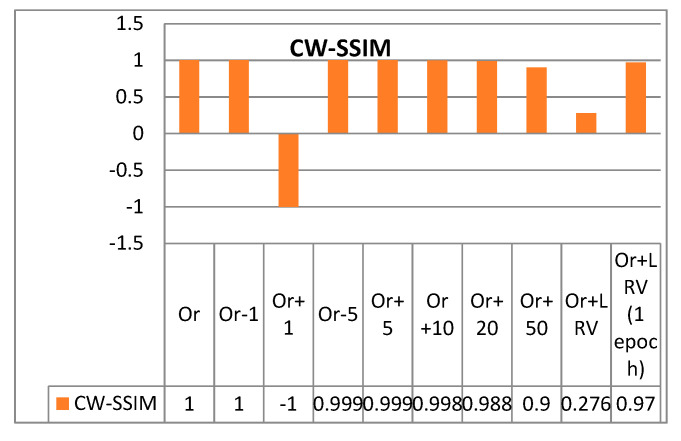
The effect of variation on CW-SSIM.

The CW-SSIM measurements are fairly stable for different variations (Figure 15). The degradation becomes more apparent for higher variations as well as for the model that uses LRV. Such a model is feasible in practice because PHM systems are likely to fall under the LRV model. This is because the noise level experienced by the system can be affected by the location of the patient as well as the condition of the sensors.

The CW-SSIM calculation in the BeCoS approach is similar to the LRV−based model, which explains why the values are lower than the CW-SSIM values for the BSBL-BO approach even though the reconstructed signals look similar. The CW-SSIM was used for the analysis in this research because it is currently regarded as one of the most robust approaches for measuring structural similarity. However, in the future, it may be necessary for researchers to develop a new type of structural similarity index that is more robust to variations such as LRV.

### 4.6. A Combination of BeCoS and BSBL-BO for Personalized Health Monitoring

The following points succinctly describe how BeCoS relates to traditional CS techniques used for the telemonitoring of biosignals:

- BeCoS can be used as a lightweight approach for significantly sparsifying an otherwise nonsparse signal. This study obtained a compression ratio of at least 75% in each case. After this stage, the sparse biosignal can be suited to traditional CS techniques
- BeCoS can be used as a standalone technique as an alternative to conventional CS techniques used for the telemonitoring of “costly-to-sparsify” biosignals.

## 5. Discussion and Conclusions

PHM of neural signals has grown in popularity, in part, due to the rising number of people with neurological disorders and the dwindling budget for healthcare. However, the utility of these systems depends on their reliability and their ability to support lightweight processing.

The sensing, compression, and reconstruction of neural signals incur a heavy penalty in terms of metrics such as energy, memory, and processing time, affecting the system’s capacity for lightweight processing. Similarly, neural signal processing relies on the frequency content of the signals and the physicians need to ensure that the processed signals have sufficient structural similarity to the original signals.

We have presented a new approach, BeCoS, for the sensing, transmission, and reconstruction of physiological signals used in PHM systems; it supports both lightweight processing and system reliability. Our approach creates a set of signals that are sensed as a derivative of a chosen signature signal. This creates a rapidly sensed pseudo-sparse signal that is a highly compressed representation of the original signal. We have compared BeCoS to the popular BSBL-BO approach for the monitoring of neural signals.

BeCoS produces signals that are translated and scaled with respect to the original signal, leading to a small degradation in the structural similarity of the reconstructed signal when compared to the original signal. However, our approach provides better results than BSBL-BO in other areas.

BeCoS can be used to enhance compressive sensing techniques for signals that are difficult to sparsify. It can also be used as a standalone approach for biosignal telemonitoring.

The results show that BeCoS is a promising approach for the sensing, transmission, and reconstruction of neural signals because it is lightweight (in terms of latency, compression ratio, and power) and is also reliable (in terms of coherence and structural similarity).

## Figures and Tables

**Table 1:** Comparison of block sparse Bayesian learning-bound optimization (BSBL-BO) and bioinspired electroceptive compressive sensing (BeCoS) coherence values.

BSBL-BO	BeCoS	% Increase
0.701	0.949	35.38%

**Table 2:** Comparison of BSBL-BO and BeCoS compression ratios (CRs).

BSBL-BO	BeCoS	% Increase
50%	76.63%	53.26%

**Table 3:** Comparison of the power and FPGA resource utilization for the BSBL-BO and BeCoS core engines.

	FFs	LUTs	IOBs	BRAMs	Power
Total number of FPGA resources	69,120	69,120	640	148	–
BSBL-BO (% utilization)	3981 (5.8%)	1743 (2.52%)	51 (7.97%)	2 (1.35%)	1.204 W
BeCoS (% utilization)	123 (0.18%)	881 (1.27%)	52 (8.13%)	3 (2.03%)	1.191 W

**Table 4:** Comparison of BSBL-BO and BeCoS complex-wavelet structural similarity (CW-SSIM).

BSBL-BO	BeCoS	% Increase
0.969	0.908	−6.295%

**Table 5:** Effect of numerical methods on signal reconstruction.

Original	Num Diff	Num Int	Num Int + Offset	Variation
40	1.2	0	40	0
41.2	1	1.1	41.1	0.1
42.2	1	2.1	42.1	0.1
43.2	0.8	3	43	0.2
44	0.8	3.8	43.8	0.2
44.8	0.6	4.5	44.5	0.3
45.4	0.6	5.1	45.1	0.3
46	0.5	5.65	45.65	0.35
46.5	0.3	6.05	46.05	0.45
46.8	0.3	6.35	46.35	0.45
47.1	0.2	6.6	46.6	0.5
47.3	0	6.7	46.7	0.6
47.3	0	6.7	46.7	0.6
47.3	−0.1	6.65	46.65	0.65
47.2	−0.2	6.5	46.5	0.7
47	−0.3	6.25	46.25	0.75
46.7	−0.5	5.85	45.85	0.85
46.2	−0.5	5.35	45.35	0.85
45.7	−0.6	4.8	44.8	0.9
45.1	−0.7	4.15	44.15	0.95
44.4	−0.8	3.4	43.4	1
43.6	−0.9	2.55	42.55	1.05
42.7	−1	1.6	41.6	1.1
41.7	−1.1	0.55	40.55	1.15
40.6	−1.2	−0.6	39.4	1.2
39.4	−1.3	−1.85	38.15	1.25
38.1	−1.4	−3.2	36.8	1.3
36.7	−1.5	−4.65	35.35	1.35
35.2	−1.6	−6.2	33.8	1.4
33.6	−1.7	−7.85	32.15	1.45
31.9	−1.8	−9.6	30.4	1.5
30.1	−1.9	−11.45	28.55	1.55
28.2	−2	−13.4	26.6	1.6
26.2	−2	−15.4	24.6	1.6
24.2	−2.2	−17.5	22.5	1.7
22	−2.3	−19.75	20.25	1.75
19.7	−2.4	−22.1	17.9	1.8
17.3	−2.5	−24.55	15.45	1.85
14.8	−2.5	−27.05	12.95	1.85
12.3	−2.7	−29.65	10.35	1.95
9.6	−2.8	−32.4	7.6	2
6.8	−2.8	−35.2	4.8	2
4	−3	−38.1	1.9	2.1
1	−3.1	−41.15	−1.15	2.15
−2.1	−3.1	−44.25	−4.25	2.15
−5.2	−3.3	−47.45	−7.45	2.25
−8.5	−3.3	−50.75	−10.75	2.25
−11.8	−3.5	−54.15	−14.15	2.35
−15.3	−3.5	−57.65	−17.65	2.35
−18.8	−3.7	−61.25	−21.25	2.45
